# Modulation of copper deficiency responses by diurnal and circadian rhythms in *Arabidopsis thaliana*

**DOI:** 10.1093/jxb/erv474

**Published:** 2015-10-29

**Authors:** Ana Perea-García, Amparo Andrés-Bordería, Sonia Mayo de Andrés, Amparo Sanz, Amanda M. Davis, Seth J. Davis, Peter Huijser, Lola Peñarrubia

**Affiliations:** ^1^Departament de Bioquímica i Biologia Molecular, Universitat de València, Av. Doctor Moliner, 50, ES-46100 Burjassot, Valencia, Spain; ^2^Departament de Biologia Vegetal, Universitat de València, Av. Doctor Moliner, 50, ES-46100 Burjassot, Valencia, Spain; ^3^Department of Comparative Development and Genetics, Max Planck Institute for Plant Breeding Research, Carl-von-Linné-Weg 10, D-50829 Cologne, Germany; ^4^Department of Biology, University of York, UK; * Present address: IIB-INTECh UNSAM-CONICET CC 164 (7130), Chascomús, Argentina.; ^†^ Present address: Unidad de Genética y Diagnóstico Prenatal, Hospital Universitario y Politécnico La Fe,Av. Campanar 21, ES-46009 Valencia, Spain.

**Keywords:** *Arabidopsis thaliana*, circadian clock, copper deficiency, copper transport, diurnal rhythm, heavy metals.

## Abstract

Copper homeostasis under deficiency is regulated by the SQUAMOSA PROMOTER BINDING PROTEIN-LIKE7 (SPL7) transcription factor. The daily oscillating expression of two SPL7-dependent copper deficiency markers, *COPPER TRANSPORTER* (*COPT2*) and *IRON SUPEROXIDE DISMUTASE* (*FSD1*), has been followed by quantitative PCR and in *promoter:LUCIFERASE* transgenic plants. Both genes showed circadian and diurnal regulation. Under copper deficiency, their expression decreased drastically in continuous darkness. Accordingly, total copper content was slightly reduced in etiolated seedlings under copper deficiency. The expression of *SPL7* and its targets *COPT2* and *FSD1* was differently regulated in various light signalling mutants. On the other hand, increased copper levels reduced the amplitude of nuclear circadian clock components, such as *GIGANTEA* (*GI*). The alteration of copper homeostasis in the *COPT1* overexpression line and *spl7* mutants also modified the amplitude of a classical clock output, namely the circadian oscillation of cotyledon movements. In the *spl7* mutant, the period of the oscillation remained constant. These results suggest a feedback of copper transport on the circadian clock and the integration of rhythmic copper homeostasis into the central oscillator of plants.

## Highlight

Cyclic expression of copper transport and the responses to copper deficiency are integrated into the light and circadian–oscillator signalling in plants.

## Introduction

In higher plants, the transition metals copper (Cu) and iron (Fe) exhibit redox properties that make them suitable to function as essential cofactors at the electronic transport chains in chloroplasts and mitochondria. Light/dark cycles result in daily changes in photosynthesis and respiration, which imply the fluctuation in the total metal requirements during the diurnal cycles and in the relative needs of the organelles (Ravet and Pilon, 2013; Yruela, 2013). The understanding of the temporary regulation of metal homeostasis and its integration with diurnal and circadian cyclic processes is starting to be elucidated. While light signalling is the main factor influencing daily changes in gene expression, the self-sustainable circadian clock generates endogenous rhythms that persist under constant conditions (Bujdoso and Davis, 2013). Among others, movement of cotyledons and leaves, hypocotyl growth, the opening and closing of flowers, the chloroplasts’ subcellular localization, and biotic and abiotic stress responses are all processes controlled by circadian rhythms in plants (Haydon *et al.*, 2013; Kinmonth-Schultz *et al.*, 2013; Sanchez and Yanovsky, 2013; Spoel and van Ooijen, 2013).

A model proposed for the Arabidopsis central oscillator includes morning and evening loops in a complex network of interlocked transcription–translation feedback loops (McClung, 2011; Bujdoso and Davis, 2013; Hsu and Harmer, 2013; Staiger *et al.*, 2013). Day–night cycles entrain circadian clock oscillations. One of the mechanisms of how the light signal is integrated in the clock is based on the interactions of the GIGANTEA (GI) protein with light, oxygen, or voltage (LOV) and F-box domain proteins that differentially regulate its stability throughout the day (Kim *et al.*, 2007; Sawa *et al.*, 2007). The interactions are triggered by blue light and stabilize GI, which contributes to amplified expression of the clock protein TIMING OF CAB EXPRESSION1 (TOC1), providing clock robustness (Kim *et al.*, 2007).

Different metals distinctly affect circadian clock parameters such as the amplitude, the phase, and the period. Under copper (Cu) deficiency, the amplitude in the oscillatory expression of two of the main components of the central oscillator, *CIRCADIAN CLOCK ASSOCIATED1* (*CCA1*) and *LATE ELONGATED HYPOCOTYL* (*LHY*), increases but their period remains mostly unaffected (Andrés-Colás *et al.*, 2010). Magnesium (Mg) deficiency also alters the amplitude of the expression of circadian clock genes (Hermans *et al.*, 2010), affecting the robustness of the clock oscillations. However, Fe deficiency extends the oscillation period of key circadian clock components in Arabidopsis plants (Chen *et al.*, 2013; Hong *et al.*, 2013; Salome *et al.*, 2013). This period lengthening provoked by Fe deficiency is considered to have a more relevant effect on the circadian clock since, linked to Fe transport, it could form an interconnected loop with the central oscillator (Chen *et al.*, 2013; Hong *et al.*, 2013; Salome *et al.*, 2013; Tissot *et al.*, 2014).

Around 90% of Arabidopsis genes exhibit daily oscillation patterns in gene expression, and at least 30% of the transcriptome is regulated by the circadian clock (Michael *et al.*, 2008). Among them, a wide range of nutrient transporters such as ATPases (HEAVY METAL ATPase; HMA1 and HMA6/PAA1) and the transporters NRAMP (NATURAL RESISTANCE-ASSOCIATED MACROPHAGE PROT EIN; NRAMP4), YSL (YELLOW STRIPE-LIKE; YSL1, YSL2, and YSL5), ZIP (ZINC TRANSPORTER PRECURSOR; ZTP29 and ZIP11), and COPT (COPPER TRANSPORTERS; COPT1 and COPT2), some of them transporting Cu, are controlled by the circadian clock (Haydon *et al.*, 2011). The daily oscillating nature of the expression of multiple metal transporters affects the circadian clock, integrating temporary information to optimize metabolism and physiology (Ko *et al.*, 2010; Sanchez *et al.*, 2011; Haydon *et al.*, 2011, 2013).

In response to Cu deficiency, eukaryotic organisms activate the expression of the high-affinity Cu transporter family CTR, whose members mediate the transport of Cu^+^ toward the cytosol. In Arabidopsis, these transporters are known as COPTs and they form a family of six members differentially regulated by Cu. Whereas plasma membrane-located members COPT1, COPT2, and COPT6 are induced by Cu deficiency at the transcriptional level, COPT3, COPT4, and COPT5 are not regulated by Cu status (Sancenón *et al.*, 2003).

The SQUAMOSA PROMOTER BINDING PROTEIN-LIKE7 (SPL7) transcription factor (TF) is considered a central component in the regulation of Cu deficiency responses (Yamasaki *et al.*, 2009; Bernal *et al.*, 2012; Zhang *et al.*, 2014). By its binding to GTAC motifs in their promoter regions, SPL7 mediates an increased incorporation of exogenous Cu by up-regulating *COPT1*, *COPT2*, and *COPT6*, genes encoding high-affinity COPT transporters (Yamasaki *et al.*, 2009; Garcia-Molina *et al.*, 2013; Perea-García *et al.*, 2013). The *spl7* mutant does not induce these and other target genes, such as *ZIP2* and *YSL2*, or different Cu-miRNAs (Burkhead *et al.*, 2009; Pilon *et al.*, 2009; Yamasaki *et al.*, 2009; Bernal *et al.*, 2012; Andrés-Colás *et al.*, 2013) in response to Cu deficiency. A self-regulatory effect of Cu on the expression of its own transporters has been hypothesized to cause an oscillation in the intracellular Cu concentration (Peñarrubia *et al.*, 2010). SPL7-mediated Cu deficiency stress perception has recently been suggested to take place at both the nucleocytoplasm and the lumen of secretory pathway compartments, rendering SPL7 as a crucial Cu sensor molecule in the two topologically different spaces where Cu is initially distributed (Garcia-Molina *et al.*, 2014). In this way, and since free Cu^+^ levels are extremely low in the cytosol (Rae *et al.*, 1999), especially under Cu-limiting conditions, cycling Cu uptake through COPT oscillations could lead to rhythmic daily changes in Cu levels that could take place in the lumen of the secretory pathway compartments.

SPL7 triggers the substitution of metalloproteins such as the Cu/Zn superoxide dismutases CSD1 and CSD2, which are replaced by the Fe counterpart FSD1 in order to make the scarce Cu available to plastocyanin (Yamasaki *et al.*, 2007). This substitution is mediated by the GTAC motifs present in the *FSD1* and *MIR398* promoter regions, where this latter miRNA mediates the degradation of *CSD* genes and their chaperone *CCS* mRNAs (Abdel-Ghany *et al.*, 2005; Pilon *et al.*, 2006). It has recently been shown that light and Cu signalling work together for optimal plant development through the interaction of the ELONGATED HYPOCOTYL5 (HY5) and SPL7 TFs at the promoters of a cohort of genes, such as those involved in anthocyanin accumulation and photosynthesis (Zhang *et al.*, 2014). In addition, HY5 also interacts physically with the core clock component CCA1 (Andronis *et al.*, 2008). Thus, the basic helix–loop–helix (bHLH) TF HY5 has been shown to integrate the light, clock, and hormonal signalling pathways (Cluis *et al.*, 2004; Vandenbussche *et al.*, 2007). While SPL7 has been shown to act as a master regulator in Cu deficiency responses (Yamasaki *et al.*, 2009, Bernal *et al.*, 2012; Zhang *et al.*, 2014), HY5 affects Cu homeostasis through the co-regulation of *miR408* (Zhang *et al.*, 2014).

In this study we examined the temporal aspects of Cu homeostasis by studying two SPL7-dependent Cu deficiency markers, *COPT2* and *FSD1*, to gain further insight into the influence of timing on plant nutrition. We also used a *COPT1* overexpression line (*COPT1* ^*OE*^) and *spl7* mutants to examine how deregulated Cu homeostasis affects circadian rhythm outputs.

## Materials and methods

### Plant growth conditions and treatments

*Arabidopsis thaliana* plants used in all experiments were of the Columbia (Col-0) ecotype, except those described in Supplementary Fig. S4 available at *JXB* online, where the ecotype Landsberg *erecta* (*L*er) was used. Plants were grown on plates as previously described (Andrés-Colás *et al.*, 2010), and incubated on MS (Murashige and Skoog, 1962) medium at half concentration (1/2 MS) [phytoagar 0.8% (w/v) plus 1% sucrose (w/v) in 0.5% MES (w/v), adjusted to pH 5.7 with KOH]. In addition, MS plates contained 0.1 µM CuSO_4_, which is considered a concentration below optimal Cu supply (Abdel-Ghany *et al.*, 2005; Yamasaki *et al.*, 2009). Cu-related variation in the growing conditions used in this work were: severe Cu deficiency [1/2 MS with 50 µM bathocuproinedisulphonic acid (BCS); –Cu+BCS], Cu deficiency (1/2 MS; –Cu), Cu sufficiency (1/2 MS with 0.5 μM or 1 μM CuSO_4_, as indicated; Ctrl), and Cu excess (1/2 MS with 10 µM CuSO_4_; +Cu).

Plants were grown under neutral day conditions with a synchronized photocycle and thermocycle [12h light (L)–12h dark (D)/12h 23 °C (H)–12h 16 °C (C); LDHC] or in the absence of both (24h light/24h 23 °C; LLHH), and in darkness with or without a thermocycle (24h dark/12h 23 °C–12h 16 °C; DDHC or 24h dark–24h 23 °C; DDHH, respectively), as indicated. For the evaluation of the influence of light quality on gene expression, the plants were grown for 7 d under blue light [seven 15W fluorescent tubes (Osram), emitting light at 6100 K and filtered through blue plastic filters, with maximal transmittance at 420 nm] or under red light [five 6W LED bulbs (LEXMAN) emitting light at 3000 K and filtered through red plastic filters, with maximal transmittance at 660 nm].

### Chimeric constructs of luciferase and Cu deficiency promoter markers

The PCR products obtained with specific primers (Supplementary Table S2 at *JXB* online) from the constructs made previously in the laboratory, *PCOPT2:GUS:NOS*, *PFSD1:GUS:NOS*, and *PFSD1(4X-GTAC):GUS:NOS* in the vector *PFP101:GUS:NOS* (Andrés-Colás *et al.*, 2013), were subcloned between the *Hin*dIII restriction sites of the binary vector pPZPXomegaL+, provided by Dr Steve Kay (USC Dornsife, USA). For *PCOPT2*, a 1246bp fragment containing four GTAC motifs, and for *PFSD1*, a fragment of 1193bp containing six GTAC boxes, were subcloned as the regulatory promoter regions and fused to *LUC* using the primers indicated in Supplementary Table S2. For *PFSD1(4X-GTAC*), a fragment of 1128bp, which was the equivalent of the preceding *PFSD1*, except for the absence of a region of 65 nucleotides (389–324bp upstream of the translational initiation region, which includes four of the six GTAC boxes in the promoter region), was also subcloned and fused to *LUC* using the primers indicated in Supplementary Table S2. This promoter with the deleted region was previously obtained in the laboratory by inverse PCR techniques (Andrés-Colás *et al.*, 2013).

The C58 strain of *Agrobacterium tumefaciens* transformed with this construct was used to transform the *A. thaliana* plants by following the floral dip protocol (Clough and Bent, 1998). Homozygous transgenic plants were selected by gentamycin resistance (75mg l^–1^) and, for each of the transgenic lines used, a plant was selected based on the highest reporter gene expression.

### Bioluminescence analysis

Seedlings were grown, as previously described (Andrés-Colás *et al.*, 2010), under neutral day LDHC conditions for 6 d and were then transferred to a multiwell plate filled with 1/2 MS with 3% sucrose, either supplemented or not with CuSO_4_ or BCS, as indicated. Then 30 µl of the reaction substrate d-luciferin (Sigma) (0.42 mg ml^–1^), as previously described (Andrés-Colás *et al.*, 2010), were added to the wells and plates were sealed with Microseal B’ Film (Bio-Rad). For correct gas exchange, breathing holes were made in the film. After another day under LDHC conditions, the multiwell plates were released to free-running LLHH conditions. Bioluminescence was measured with Luminoskan Ascent, and data were processed with the Ascent software, v. 2.6 (Thermo Scientific). Experiments to measure *in vivo* luminescence in Fig. 3 were performed in microtitre plates, as previously reported (Hanano *et al.*, 2008). Here seedlings were entrained for 7 d under 12h light/12h dark intervals at 20 °C, and then released to constant light and temperature conditions (LLHH).

The experiment to assess the effect of Cu on luciferin was performed on total plant protein extracts. The reaction contained 15 μl of extract+100 μl of assay buffer (15 mM MgSO_4_, 15 mM KPO_4_ pH 7.8, 4 mM EDTA pH 7.8, 2 mM ATP, 2 mM DTT) + 50 μl of d-luciferin (0.3 mg ml^–1^) + CuSO_4_, and luciferase activity was measured for the indicated times.

### Measurement of cotyledon movement

To study circadian rhythms in Arabidopsis seedlings from the movement of cotyledons, 6-day-old wild-type (WT), *COPT1* ^*OE*^, *spl7* mutant, and *spl7* complemented with *SPL7* (*CSPL7*) seedlings (Bernal *et al.*, 2012), grown under continuous conditions, were analysed according to Salathia *et al.* (2006). Plates were first incubated in a neutral photoperiod (LDHC) for 5 d. Then seedlings were transferred to new 25-well plates (BibbySterilin Ltd, UK), which had been previously layered with 1ml of 1/2 MS medium containing 1.5% phytoagar. After 24 h of growth in chambers, plates were placed in front of a camera under continuous light and temperature (LLHH) conditions. Photographs were taken every 30 min for 6 d. The position of the cotyledons was recorded from the photographs taken of the WT and *COPT1* ^*OE*^ mutant plants at 2h intervals. Alternatively, the MetaMorph software (Southern and Millar, 2005) was used. Analysis of the clock parameters was performed with the BRASS program (http://millar.bio.ed.ac.uk).

### Gene expression analysis by RT–PCR

Total RNA was extracted with Trizol Reagent (Ambion) and reverse transcribed to cDNA with SuperScript II (Invitrogen) and p(dT)_15_ (Roche) as described (Andrés-Colás *et al.*, 2006). Semi-quantitative PCRs (sqPCRs) were performed with specific oligonucleotides (Supplementary Table S3 at *JXB* online) and the products were visualized in 1.5% agarose gels. The relative values of the mRNA levels for each sample were normalized in relation to *ACT1* amplicons as the loading control. qPCRs were composed of 12.5 µl of the Power SYBR Green Master Mix (Applied Biosystems), 15 ng of cDNA, and 0.3 µM of primers for all the genes in a 25 µl total reaction volume (Supplementary Table S4). The qPCRs were carried out in a GFX96 Touch Real-Time PCR Detection System (Bio-Rad) under conditions previously described elsewhere (Perea-García *et al.*, 2013). The relative values of the mRNA levels for each sample were normalized against transcript levels of *UBQ10*, used as the reference gene.

### Metal accumulation measurements

Cu content was determined as described (Andrés-Colas *et al.*, 2006) at the ‘Servei Central d’Instrumentació Científica (Universitat Jaume I)’. Lyophilized samples were digested with HNO_3_ at 100 ºC and diluted with H_2_O milliQ_PLUS_ (Millipore). The Cu content of each sample was determined by inductively coupled plasma mass spectrometry (ICP-MS).

### Data analysis

The analysis of gene expression under multiple cycling conditions was performed using the DIURNAL database (http://diurnal.mocklerlab.org/). The theoretical analysis of promoter sequences was conducted using the PLACE (Plant *cis*-acting regulatory DNA elements) database (http://www.dna.affrc.go.jp/htdocs/PLACE/signalscan.html) and PATMACH (http://www.arabidopsis.org/cgi-bin/patmatch/nph-patmatch.pl). For the analysis of leaf movement rhythms and bioluminescence (period, phase, and amplitude), the MS Excel Software interface Biological Rhythm Analysis System (BRASS) (Southern and Millar, 2005) was employed (available at http://millar.bio.ed.ac.uk/PEBrown/BRASS/BrassPage.htm) using the FFT-NLLS (Plautz *et al.*, 1997) algorithm and a relative amplitude error (RAE) ≤0.5. The statistical analysis of relative expression was performed by comparing the relative expression of the genes (RT–PCR) based on a comparison in pairs fixed on a random test reallocation (*P*<0.05) (Pfaffl *et al.*, 2002). For the other parameters, a two-way analysis of variance (ANOVA) with a Duncan test (*P*≤0.05) or a *t*-test for Cu content was performed using the InfoStat software, version 2010 (http://www.infostat.com.ar).

## Results

### Kinetics of expression of Cu deficiency markers at different Cu levels

**Fig. 1.**
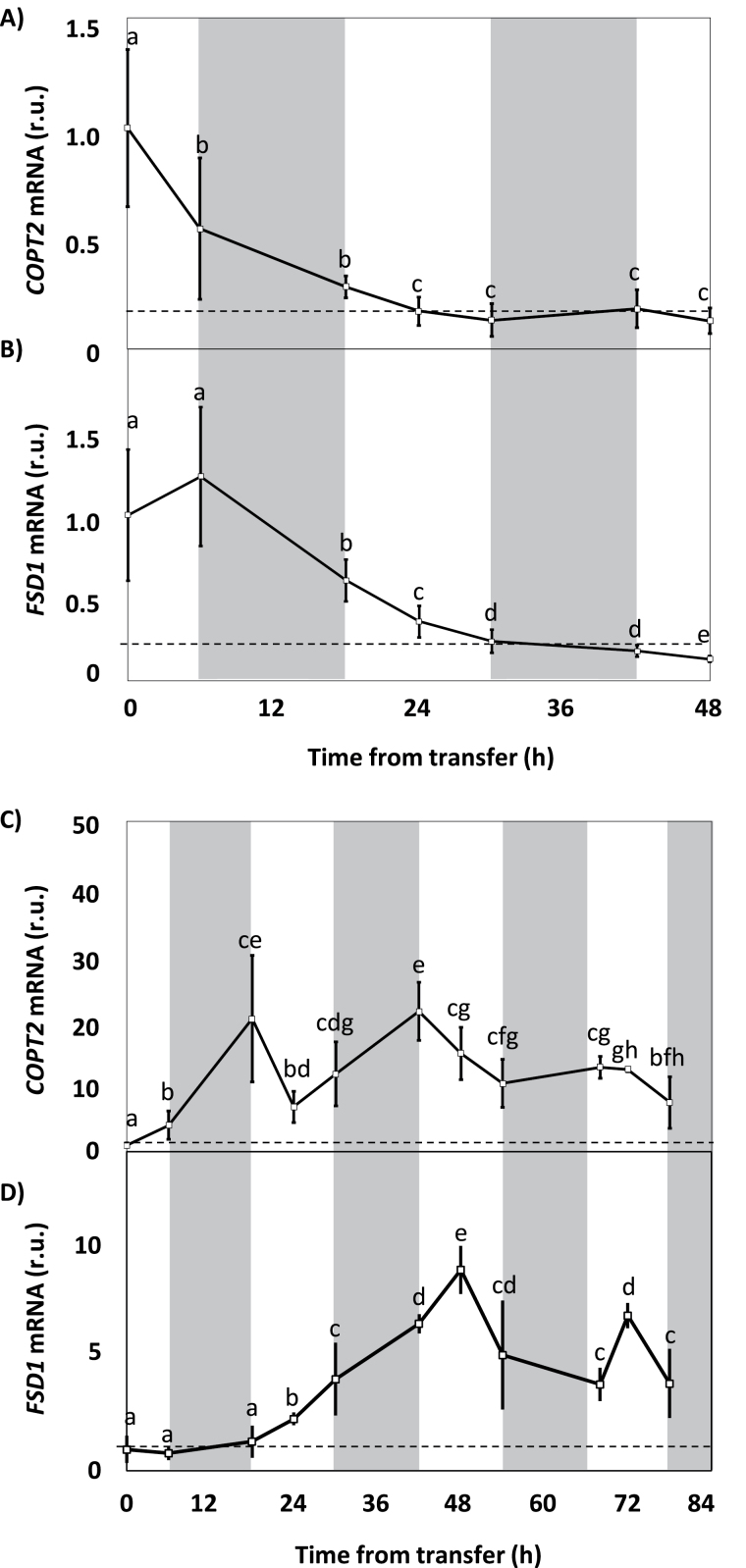
Kinetics of *COPT2* and *FSD1* gene expression following the transfer from severe Cu deficiency to sufficiency, and vice versa. (A) *COPT2* and (B) *FSD1* gene expression in 6-day-old WT seedlings grown in severe Cu deficiency (1/2 MS with 50 µM BCS) and transferred to Cu sufficiency (1/2 MS with 1 µM CuSO_4_). (C) *COPT2* and (D) *FSD1* gene expression in 6-day-old seedlings grown in Cu sufficiency (1/2 MS with 1 µM CuSO_4_) and transferred to severe Cu deficiency (1/2 MS with 50 µM BCS). Samples were collected at the time of transfer (0 time on the *x*-axis) and then at 0, 6, and 12 h from the start of the light phase (light, white; dark, grey) for 2 d in (A) and (B) or 3.5 d in (C) and (D). The mRNA was analysed by qPCR with specific primers and normalized to *UBQ10* gene expression. mRNA levels are expressed as relative units (r.u.) and referred to the sample at time zero (transfer time). Each point represents the mean ±SD of three replicates. Means with a different letter are significantly different (*P*<0.05).

In order to determine the time required to perceive changes in the environmental levels of Cu in Arabidopsis seedlings, the gene expression kinetics of the metal deficiency markers *COPT2* and *FSD1* were evaluated in seedlings grown under 12h light and temperature cycles (LDHC) and different Cu regimes: sufficiency (1/2 MS with 1 µM CuSO_4_) and severe deficiency (1/2 MS with 50 µM BCS) for 6 d, after which they were transferred to media under the opposite Cu condition (from sufficiency to severe deficiency and from severe deficiency to sufficiency) (Fig. 1). After the initial sample was collected at time zero before transfer, sampling for qPCR gene expression analysis took place at 0, 6, and 12h of the light period for 2–3 d. The light/dark periods are shown in Fig. 1 in white and grey backgrounds, respectively. The seedlings grown under severe Cu deficiency and then moved to the sufficiency condition showed a relative decrease in *COPT2* (Fig. 1A) and *FSD1* (Fig. 1B) expression. The relative expression of both genes was stabilized when a reduction of ~80% was reached with respect to the time zero expression. However, the repression kinetics differed depending on the gene evaluated. A 50% repression was observed for *COPT2* 6h after transfer (Fig. 1A), while a half-repression was obtained for *FSD1* some 18h later (Fig. 1B).

When seedlings were grown under Cu-sufficient conditions and transferred to severe Cu deficiency, the expression of both genes *COPT2* and *FSD1* was induced (Fig. 1C, D). While the *COPT2* expression increased 20-fold 18h after transfer (Fig. 1C), *FSD1* showed the maximum increase (~10-fold) 30h later (Fig. 1D), which confirmed the differences in the timing of the response to Cu changes between *COPT2* and *FSD1* (Fig. 1A, B). After the initial increase, the expression was not stabilized once it reached its maximum, and oscillations were observed in both cases during an ~24h period. Indeed the maximal expression of both genes occurred at different cycle time points, at 0h and 6h, respectively (Fig. 1C, D).

### Cyclic expression patterns of Cu deficiency markers

**Fig. 2.**
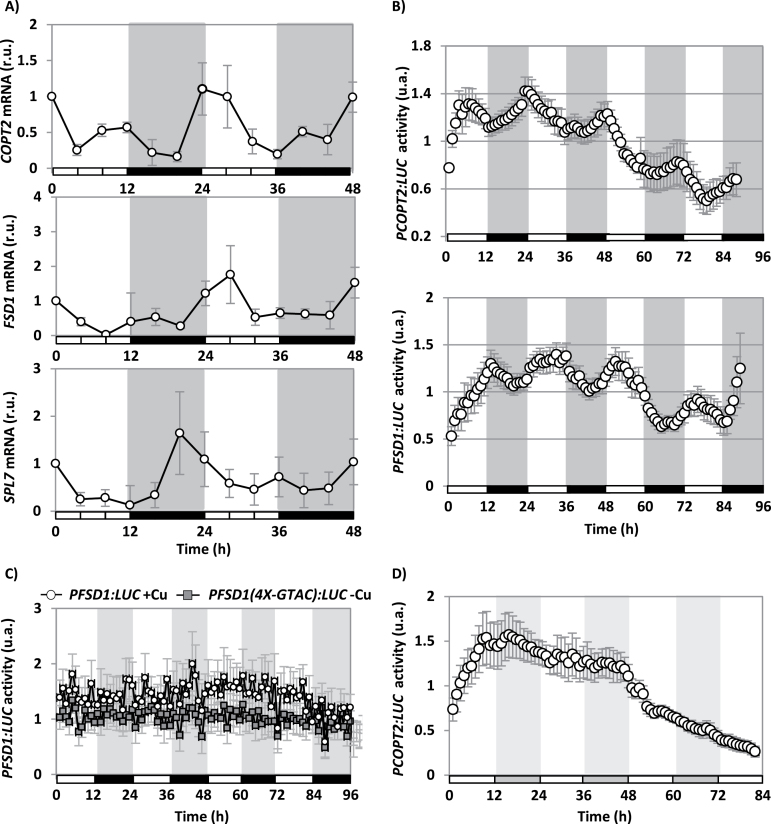
Diurnal *COPT2*, *FSD1*, and *SPL7* gene expression in Cu deficiency. (A) WT seedlings were grown for 6 d under Cu deficiency (1/2 MS) in LDHC conditions. Samples were collected every 4 h during 2 d. The *COPT2*, *FSD1*, and *SPL7* relative expression was analysed by qPCR. The *UBQ10* gene was used as reference. The values are the mean of two replicates. mRNA levels are expressed as relative units (r.u.). (B) Bioluminescence of the *PCOPT2:LUC* and *PFSD1:LUC* seedlings grown under severe Cu deficiency (1/2 MS with 50 µM BCS), entrained for 7 d. Bioluminescence was measured under LDHC conditions every hour. The values are the mean ±SEM of 6–11 replicates. The grey and white bars indicate night and day, respectively. (C) Bioluminescence of the *PFSD1:LUC* and *PFSD1*(*4X-GTAC):LUC* seedlings grown under Cu deficiency (1/2 MS; grey squares) or Cu excess (1/2 MS with 10 µM CuSO_4_; white circles) entrained for 7 d. Bioluminescence was measured every hour. The values are the mean ±SEM of 6–12 replicates. The grey and white bars indicate the day and night, respectively. (D) Bioluminescence of the *PCOPT2:LUC* seedlings grown under Cu deficiency (1/2 MS) entrained for 7 d in LDHC and transferred to DDHH conditions. Bioluminescence was measured every hour after transfer to DDHH conditions. The values were the mean ±SEM of six replicates. The grey and white bars indicate the subjective day and night, respectively. The values are normalized to the mean of each trace over the time course and are expressed as arbitrary units (a.u.).

The *FSD1*, *COPT2*, and *SPL7* expression during the diurnal cycle was studied by qPCR under Cu deficiency (1/2 MS) (Fig. 2A). Our data suggest that the expression of *COPT2*, *FSD1*, and *SPL7* was not constant but oscillated during subsequent light/dark periods, showing maximal values at different times. Thus, *COPT2* expression showed a peak at ~0h and *FSD1* at ~6h, while *SPL7* expression oscillated in advance with a peak at ~18–22h (Fig. 2A). Under Cu excess conditions (1/2 MS with 10 µM CuSO_4_), gene expression decreased drastically, to the extent that oscillation was mostly abolished for *COPT2* and *FSD1* (not shown).

To study the continuous expression of the SPL7 target genes in response to Cu deficiency, *LUC* reporter transgenes driven by either *COPT2*- or *FSD1*-derived promoters (*PCOPT2:LUC* and *PFSD1:LUC*, respectively) were constructed, and transformed into Arabidopsis WT plants as described in the Materials and methods. In order to determine whether addition of Cu affects luciferase activity, we used *PLHY:LUC* transgenic plants (Roden *et al.*, 2002). To that end, total protein extracts were obtained from the *PLHY:LUC* seedlings cultured for 7 d in Cu deficiency (1/2 MS) and Cu excess (1/2 MS with 10 µM CuSO_4_) (Supplementary Fig. S1 at *JXB* online). The seedlings grown in Cu deficiency were used as a positive luciferase activity control, and the extract without d-luciferin substrate was employed as a negative control. From the obtained results (Supplementary Fig. S1), it was concluded that Cu excess at 10 μM Cu does not affect the assay. Next we checked whether the expression of *LUC* in the transgenic plants reproduced the expression pattern of the endogenous gene. To this end, *PFSD1:LUC* and *PCOPT2:LUC* seeds were cultured for 6 d under severe Cu deficiency (1/2 MS with 50 μM BCS), deficiency (1/2 MS), sufficiency (1/2 MS with 1 μM CuSO_4_), and excess (1/2 MS with 10 μM CuSO_4_). RNA was extracted from the seedlings and *LUC* expression was analysed by sqPCR with specific primers (Supplementary Fig. S2). The *COPT2* and *FSD1* relative gene expression was used as a control of Cu deficiency regulation, and *ACT1* gene expression was used as the RNA loading control. The relative expression of *LUC*, *COPT2*, and *FSD1* in transgenic plants *PCOPT2:LUC* and *PFSD1:LUC* is shown in Supplementary Fig. S2. In all cases, *LUC* expression was regulated by the Cu concentration in the medium, similarly to the endogenous control genes *COPT2* and *FSD1*, thereby validating the obtained transgenic plants for studying the continuous response to Cu deficiency.

*PCOPT2:LUC* and *PFSD1:LUC* seedlings were grown under severe Cu deficiency, and luciferase activity was continuously recorded under a neutral photoperiod (LDHC). As shown in Fig. 2B, luciferase activity in both *PCOPT2:LUC* and *PFSD1:LUC* seedlings oscillated with an ~24h period. Furthermore, the timing of maximal activity differed between the *PCOPT2:LUC* seedlings, whose activity peaked in the morning (~0h), and for the *PFSD1:LUC* seedlings, whose maximum peaked at mid-day (~6h). In order to determine the interaction between the Cu-responsive elements (CuREs) and the diurnal oscillation, *PFSD1(4X-GTAC):LUC* seedlings, which lacked four of the six GTAC boxes found in the vicinity of the transcriptional start site (see the Materials and methods), were also assayed under the same conditions (Fig. 2B). It is noteworthy that these seedlings completely lost induction under Cu deficiency and behaved like the *PFSD1:LUC* seedlings cultured under Cu excess conditions (Fig. 2C). On the other hand, when *PCOPT2:LUC* seedlings were maintained in darkness, the luciferase activity greatly diminished after 48h (Fig. 2D), which suggests that light plays an important role in Cu deficiency-mediated responses.

**Fig. 3.**
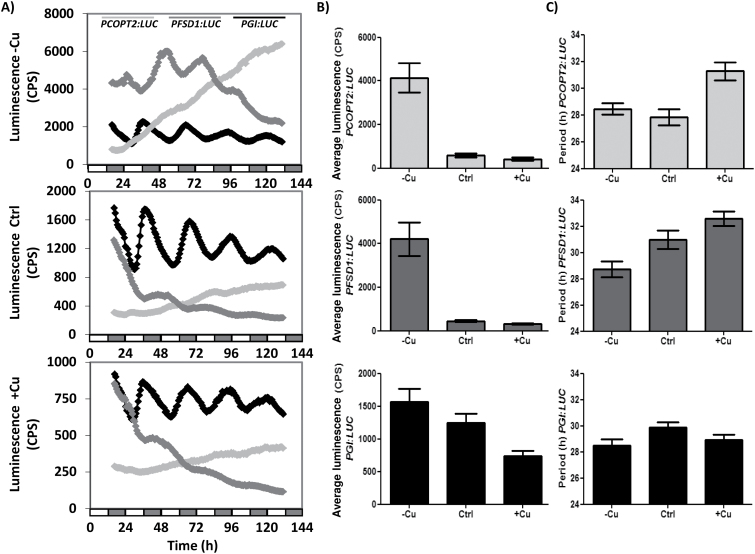
Luciferase activity driven by the *COPT2* and *FSD1* promoters. (A) Bioluminescence of the *PCOPT2:LUC* (grey line), *PFSD1:LUC* (dark grey line), and *PGI:LUC* (black line) seedlings grown under Cu deficiency (1/2 MS; –Cu), Cu sufficiency (1/2 MS with 1 µM CuSO_4_; Ctrl), or Cu excess (1/2 MS with 10 µM CuSO_4_; +Cu) entrained for 7 d in 12h light/12h dark at 20 °C and transferred to constant light conditions. Bioluminescence was then measured every hour. The values are the mean ±SEM of replicates (*n*=12). The grey and white bars indicate the subjective night and day, respectively. (B) Average luminescence of the *PCOPT2:LUC* (grey), *PFSD1:LUC* (dark grey), and *PGI:LUC* (black) seedlings grown on the same conditions as in (A). (C) Period of the *PCOPT2:LUC* (grey), *PFSD1:LUC* (dark grey), and *PGI:LUC* (black) seedlings grown on the same conditions as in (A).

Bioluminescence oscillations caused by changes in the expression of the chimeric genes *PCOPT2:LUC* and *PFSD1:LUC* were also followed in comparison with those of *PGI:LUC*, under different Cu regimes: Cu deficiency (1/2 MS; –Cu), sufficiency (1/2 MS with 1 µM CuSO_4_; Ctrl), and excess (1/2 MS with 10 µM CuSO_4_; +Cu) in constant conditions of light and temperature (LLHH) (Fig. 3). The rhythmical pattern shown by *PGI:LUC* was clearly observed under the three Cu regimes used (Fig. 3A) and, although periodicity was not affected, the average luminescence decreased with increasing Cu levels (Fig. 3B). Oscillations of *PCOPT2:LUC* and *PFSD1:LUC* were also dependent on Cu levels. In both cases, not only did the average luminescence decrease drastically in the presence of Cu, but oscillation periodicity was also affected. Thus, in *PFSD1:LUC* the period increased gradually with the Cu level, while in *PCOPT2:LUC* the period increased only in the presence of Cu excess (Fig. 3B).

### Influence of light on the response of the cycling genes to Cu deficiency

**Fig. 4.**
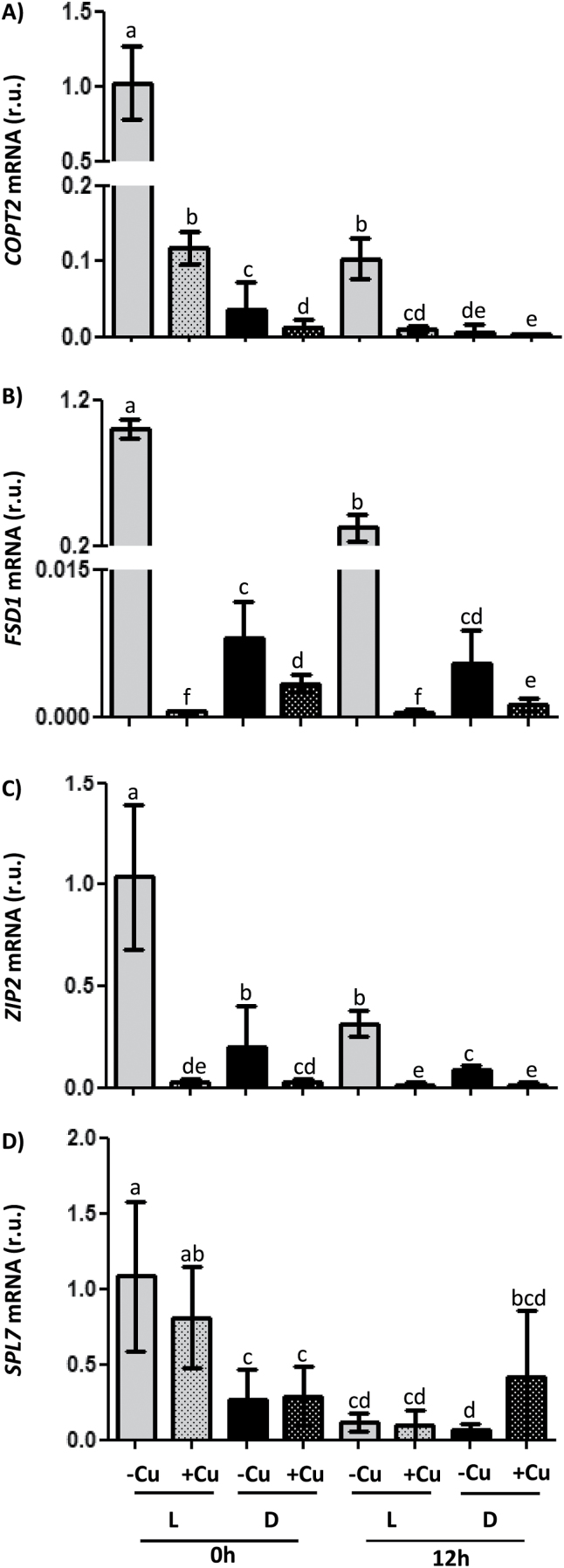
Regulation of *COPT2*, *FSD1*, *ZIP2*, and *SPL7* gene expression by Cu and light conditions. The relative expression of the (A) *COPT2*, (B) *FSD1*, (C) *ZIP2*, and (D) *SPL7* genes was determined by qPCR in 7-day-old WT seedlings grown under Cu deficiency (1/2 MS; –Cu, plain bars) or Cu excess (1/2 MS with 10 µM CuSO_4_; +Cu, stippled bars), in LDHC (L, grey bars) or DDHC (D, black bars). Samples were collected at 0h and 12h. The *UBQ10* gene was used as reference. mRNA levels are expressed as relative units (r.u.). The bars represent the mean ±SD of three replicates. Means with a different letter are significantly different (*P*<0.05).

To assess the interaction of light and the response to Cu deficiency, WT seedlings were grown for 7 d in a neutral photoperiodic regime, under LDHC conditions or in darkness with temperature cycles (DDHC, etiolated plants), and, in both cases, under Cu deficiency (1/2 MS; –Cu) and Cu excess (1/2 MS with 10 µM CuSO_4_; +Cu). Samples were collected at 0h and 12h and then the relative expression of markers known to be rapidly induced by Cu deficiency was analysed by qPCR. These markers included *COPT2*, *FSD1*, *ZIP2*, and *SPL7*, and, as expected, the expression of *COPT2, FSD1*, and *ZIP2* was induced by Cu deficiency under the LDHC conditions at either 0 or 12h, with transcript levels being higher at 0h than at 12h in all three cases (Fig. 4). Compared with LDHC, ~96% of the *COPT2* expression in Cu deficiency was lost in DDHC at 0h (Fig. 4A), when the *COPT2* expression peaked (Figs 1, 2, 3A). Similar effects of light were also observed for the expression of *FSD1* and *ZIP2* (Fig. 4B, C). It is noteworthy that even though differences in relative expression were much smaller in etiolated seedlings, induction under Cu deficiency was still significant. However, *SPL7* expression remained unaffected by Cu and, at least at 12h, also by darkness, whereas a minor effect of light is observed at 0h. Moreover, and as expected since it was mediated by miR398 degradation, Cu excess induced the expression of the *CSD1* and the *CCS* marker genes under LDHC conditions at both 0h and 12h, and expression was greater at 0h than at 12h in both cases (Supplementary Fig. S3 at *JXB* online). However, this induction in etiolated seedlings was minimal for *CSD1* (Supplementary Fig. S3A) or was not maintained at 12h for *CCS* (Supplementary Fig. S3B), and light seemed critical for responses to Cu excess.

The temporal expression patterns of genes shown in Fig. 4, and in Supplementary Fig. S3 at *JXB* online were analysed with the DIURNAL software (http://diurnal.mocklerlab.org/) (Mockler *et al.*, 2007) (Table 1; Supplementary Table S1). This software allowed us to obtain the temporal gene expression patterns and to distinguish between diurnal and circadian oscillations by comparing gene expression under multiple cycling conditions. Supplementary Table S1 shows the phase of the peak values for the Cu homeostasis genes under different cycling conditions in accordance with the data obtained from the DIURNAL database (Mockler *et al.*, 2007). Table 1 integrates different parameters for the genes used in this work and others, including the number of different conditions in which significant oscillation in transcript levels can be observed, indicated in Supplementary Table S1, and the average peak values. In addition, the number of GTAC elements in their promoter regions and the expression values (log_2_ ratio) from a previous microarray analysis comparing deficiency against excess Cu (Andrés-Colás *et al.*, 2013) are also shown (Table 1). *COPT2* displayed a higher number of cycling conditions, both diurnal and circadian, than *FSD1* (Supplementary Table S1). Curiously, Cu homeostasis genes with the highest number of cycling conditions showed average peak values at ~21.5h (Table 1), suggesting a correlation between the abundance of the transcriptional regulator SPL7 and the intensity of Cu deficiency responses.

**Table 1.** Analysis of the circadian and diurnal oscillatory behaviour of the genes regulated under Cu deficiency conditions The columns show the locus MIPS code (http://www.arabidopsis.org/), the number of GTAC motifs at the promoter regions (500bp upstream of the transcriptional start), the expression values (log_2_ ratio) obtained in a microarray analysis comparing copper deficiency and excess (Andrés-Colás *et al.*, 2013), the number of cycling conditions, and the average of the phase values at different oscillating conditions (see Supplementary Table S1 at *JXB* online) (http://diurnal.mocklerlab.org).

Gene	Locus	GTAC	Ratio	No. of cycling conditions	Peak
*COPT2*	At3g46900	4	–2.860	5	21.8
*FSD1*	At4g25100	6	–3.834	2	3.5
*SPL7*	At5g18830	0	–	4	21.5
*ZIP2*	At5g59520	5	–3.243	1	19
*CSD1*	At1g08830	2	2.496	3	0.3
*CCS*	At1g12520	1	2.642	5	21.4
*LHY*	AT1G01060	3	–1.594	11	0.2
*CAS*	AT5G23060	5	–1.497	9	0.7
*YSL2*	AT5G24380	4	–2.593	4	22.3
*SMC6B*	AT5G61460	3	–1.187	3	18.0
*IRT1*	AT4G19690	3	–1.167	3	1.0
*ACA1*	AT3G52720	2	–1.674	8	22.5
*TIR-NBS-like*	AT3G04210	2	–1.188	3	22.7
*CYP83A1*	AT4G13770	2	–1.353	8	5.4
*GSTF11*	AT3G03190	3	–1.157	4	6.0
*BCAT4*	AT3G19710	3	–1.054	5	6.2
*UGT78D2*	AT5G17050	3	–1.035	9	22.1
*EXPA8*	AT2G40610	2	–1.405	7	4.0
*Aux-response*	AT1G29440	3	–1.282	10	2.6
*COPT1*	At5G59030	4	–	4	21.5
*CCH*	AT3G56240	4	–1.819	2	22
*CSD2*	AT2G28190	0	3.410	4	17.3

**Fig. 5.**
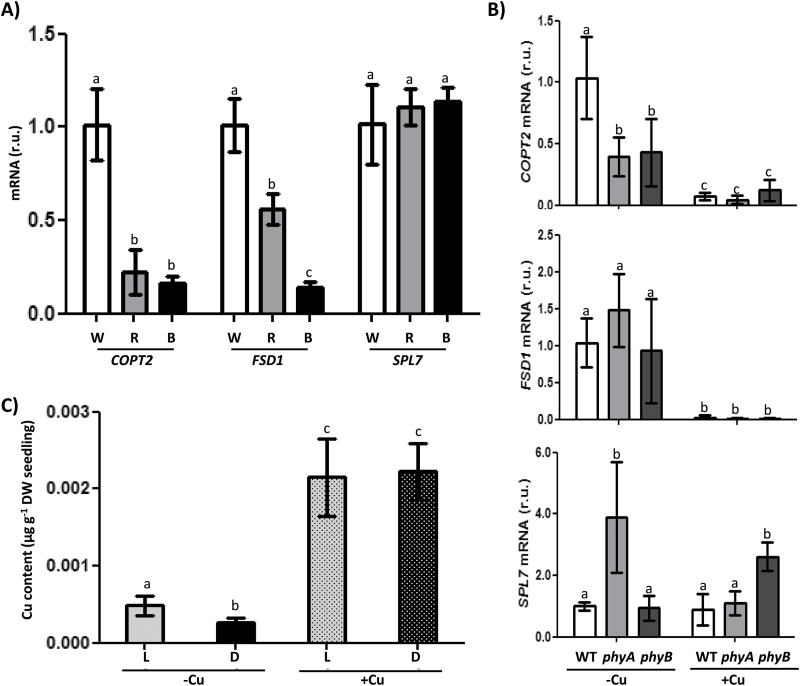
Copper content and regulation of *COPT2*, *FSD1*, and *SPL7* gene expression under different light conditions. (A) *COPT2*, *FSD1*, and *SPL7* expression in WT seedlings grown under white, red, and blue light. The relative gene expression was determined by qPCR in 7-day-old WT seedlings, grown under Cu deficiency (1/2 MS), using the *UBQ10* gene as reference. Seedlings were grown under white light (W, white bars), red light (R, grey bars), or blue light (B, black bars). (B) *COPT2*, *FSD1*, and *SPL7* expression in WT (white bars), *phyA* (grey bars), and *phyB* (dark grey) mutants under Cu deficiency (1/2 MS; –Cu) or Cu excess (1/2 MS with 10 µM CuSO_4_; +Cu). The mRNA levels are expressed as relative units (r.u.). The bars represent the mean ±SD of three replicates. (C) Endogenous Cu content in the Arabidopsis seedlings grown on different Cu-supplemented media. The Cu content from 6-day-old WT seedlings grown in Cu deficiency (1/2 MS; –Cu; plain bars) or Cu excess (1/2 MS with 10 µM CuSO_4_; +Cu; stippled bars) under LDHC (L; grey bars) or DDHC (D; black bars) conditions. Values are means ±SD of three replicates. Means with a different letter are significantly different (*P*<0.05).

In order to study light influence on Cu deficiency responses further, WT Cu-deficient plants were grown under different light types: white, red, and blue (Fig. 5A). Gene expression analyses showed that whereas *SPL7* expression was unaffected by the different type of lights, expression of two of its targets, *COPT2* and *FSD1*, was affected by light quality. Red light affected *COPT2* more than *FSD1* expression, while blue light greatly decreased the expression of both genes (Fig. 5A). Similarly, down-regulation of *COPT2* took place in the phytochrome mutants *phyA* and *phyB*, whereas *FSD1* expression remained unaffected and *SPL7* expression even increased in the *phyA* and *phyB* mutants under Cu deficiency and Cu excess, respectively (Fig. 5B).

As Cu deficiency responses were greatly attenuated in continuous darkness, we checked whether the reduced expression of the Cu transporter, *COPT2*, under Cu deficiency affected the global Cu content in etiolated seedlings. For this purpose, the Cu content per plant was determined under both Cu deficiency and excess (Fig. 5C). In agreement with *COPT2* expression attenuation, seedlings grown under Cu deficiency had a slightly but significantly lower Cu content when kept in the dark compared with when they were grown in LDHC conditions. They showed a similar Cu content under both light/dark regimes when grown in Cu excess (Fig. 5C).

### Influence of Cu status on the regulation of clock-related genes

**Fig. 6.**
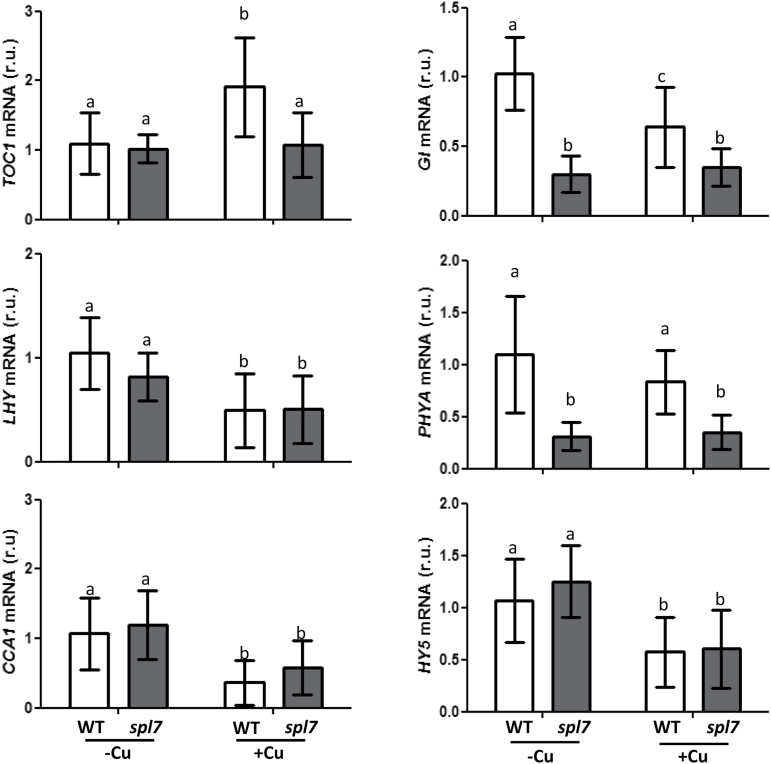
Regulation of *TOC1*, *LHY*, *CCA1*, *GI*, *PHYA*, and *HY5* gene expression in the WT and *spl7* mutant. The relative expression of the genes was determined by qPCR in 7-day-old WT (white bars) and *spl7* (dark grey bars) seedlings grown under Cu deficiency (1/2 MS; –Cu) or Cu excess (1/2 MS with 10 µM CuSO_4_; +Cu). The *UBQ10* gene was used as reference. mRNA levels are expressed as relative units (r.u.). The bars represent the mean ±SD of three replicates. Means with a different letter are significantly different (*P*<0.05).

The relative expression of the clock-related genes *TOC1*, *LHY*, *CCA1*, and *GI*, as well as of the photoreceptor gene *PHYA* and the photomorphogenesis-promoting TF gene *HY5*, was also followed under conditions of Cu deficiency (1/2 MS; –Cu) and Cu excess (1/2 MS with 10 µM CuSO_4_; +Cu), in both WT and *spl7* mutant plants (Fig. 6). With the exception of *TOC1*, clock-related genes were induced by Cu deficiency (Fig. 6). However, while *LHY* and *CCA1* mRNA levels varied in the *spl7* mutant according to the Cu regime, *GI* and *PHYA* expression levels did not increase under Cu deficiency in the mutant. Furthermore, in WT plants, *PHYA* expression was found not to be significantly affected by the Cu status, whereas the expression of *HY5* followed the same pattern as *LHY* and *CCA1*, as in both WT and *spl7* mutants mRNA levels were higher under Cu deficiency (Fig. 6).

### Cotyledon movement in COPT1^OE^ and spl7 mutants

**Fig. 7.**
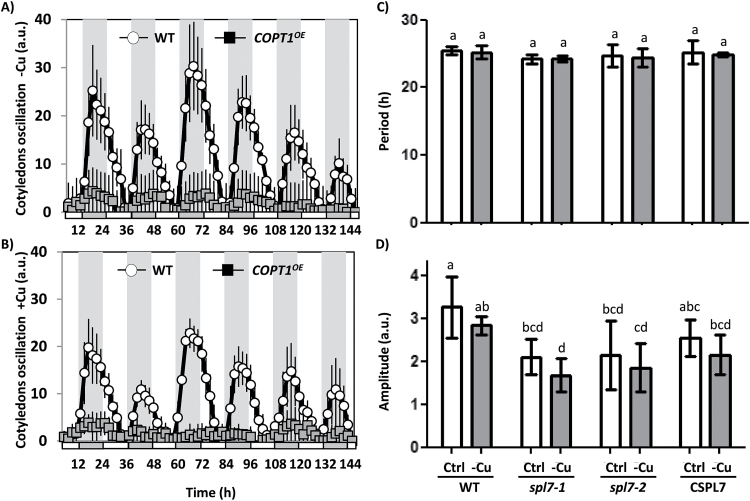
Cotyledon movement in WT, *COPT1* ^*OE*^, and *spl7* seedlings. WT and *COPT1* ^*OE*^ seedlings were grown for 5 d in (A) Cu deficiency (1/2 MS; –Cu) and (B) Cu excess (1/2 MS with 10 µM CuSO_4_; +Cu) under a neutral photoperiod. The seedlings were transferred to new plates (1/2 MS containing 1.5% phytoagar). After 24h, photographs were taken under continuous light for 6 d, every 2h. Data shown have been corrected for position changes due to plant growth and cotyledon senescence by subtracting these trends, calculated according to linear regression of the daily minimum values. Mean values ±SD of *n*=5 are shown. The subjective night is shown as grey areas. Mean periods and amplitudes of WT plants were 24.4±0.3 and 9.1±1.3 in –Cu and 24.2±0.4 and 8.3±1.4 in +Cu. (C) Period and (D) amplitude of the cotyledon movement in the *spl7* mutant. WT, *spl7-1*, *spl7-2*, and *CSPL7* (*spl7-2* line complemented with *SPL7*) seedlings were grown for 5 d in Cu sufficiency (1/2 MS with 0.5 µM Cu; Ctrl, white bars) and Cu deficiency (1/2 MS; –Cu, grey bars) and processed as in (A and B) but photographs were taken every 30 min. a.u., arbitrary units. Means with a different letter are significantly different (*P*<0.05).

Cyclic movement of cotyledons, one of the most studied clock outputs (Millar *et al.*, 1995) was examined to check Cu-related rhythm disturbances. The rhythmic behaviour of WT, *COPT1* ^*OE*^, *spl7* mutant, and *CSPL7* seedlings was studied. As shown in Fig. 7A and B, the oscillation of the cotyledon movements was slightly reduced in WT seedlings under Cu excess while in the *COPT1* ^*OE*^ mutants they almost disappeared, independently of the Cu level. The period remained unchanged for *spl7* seedlings if compared with the WT when both were grown under Cu deficiency and sufficiency (Fig. 7C). However, the amplitude of the oscillations decreased in the *spl7* lines, under both Cu sufficiency and deficiency (Fig. 7D). The complemented *CSPL7* line showed an intermediate situation between the WT and the *spl7* mutant, which indicates partial phenotype restoration (Fig. 7D). Taken together, these results support the potential interaction between the central oscillator of the circadian clock and SPL7-regulated Cu homeostasis, including *COPT2*, among other COPTs of the plasma membrane, which mediates Cu transport and might be responsive to intracellular oscillating Cu levels.

## Discussion

In this work, the temporal expression pattern of genes involved in Cu homeostasis, such as *COPT2* and *FSD1,* has been studied, including the role of both light and the circadian clock in its regulation. The *COPT2* and *FSD1* daily oscillation phases under light/dark cycles are found at 0h and 6h, respectively (Figs 1, 2A, B). Furthermore, although the amplitude was drastically reduced in both cases under continuous conditions, *COPT2* and *FSD1* were still cycling (Fig. 3), indicating that they are subjected to the circadian clock. The molecular logic behind the scheduled peaks in *COPT2* and *FSD1* expression during the light period of the cycle could be aimed at fulfilling specific physiological plant demands. Since *COPT2* expression peaks at 0h and taking into account a delay in translation, the COPT2 protein level would reach its maximum well in advance of the chloroplast’s largest Cu requirement, namely during photosynthesis involving plastocyanin as one of the most Cu-demanding proteins in plant cells (Burkhead *et al.*, 2009). On the other hand, *FSD1* expression levels are delayed (peak at 6h) which would be appropriate to deal with increased reactive oxygen species (ROS) levels, as a consequence of the electronic transport chain functioning in photosynthesis (Ravet and Pilon, 2013). Thus, light and the circadian clock, respectively, are involved in the regulation of these genes, and may contribute together with other factors to potentiate Cu deficiency responses at ~0h and to attenuate them at ~12h (Fig. 4). Consistently, Cu content is lower in etiolated versus light-grown seedlings under Cu deficiency (Fig. 5C). Remarkably, gene regulation under Cu deficiency still oscillates in etiolated seedlings (Fig. 4). Despite *COPT2* expression being greatly attenuated in relation to the LDHC conditions, it is still significantly different between 0h and 12h in DDHC (Fig. 4A). Conversely, however, Fe deficiency responses are completely dependent on light (Salome *et al.*, 2013).

SPL7 functions as a master regulator in Cu deficiency responses (Yamasaki *et al.*, 2009; Bernal *et al.*, 2012; Zhang *et al.*, 2014). Although we have not determined the continuous *SPL7* expression pattern in luciferase transgenic plants, the analysis by qPCR under dark/light cycles suggests an oscillation in its expression with a phase at ~20h (Fig. 2A). Our results are mostly in agreement with the *in silico COPT2*, *FSD1*, and *SPL7* gene expression patterns conducted with the DIURNAL software (http://diurnal.mocklerlab.org). Under continuous LLHH conditions, in the WT plants, *COPT2* peaks at 0h, *FSD1* lacks a clear oscillatory pattern, and *SPL7* shows a phase at ~20h (19–23h) (Supplementary Table S1 at *JXB* online). This was as expected as it has to be in advance of its targets.

Interestingly, *SPL7* and SPL7 targets, such as *COPT2*, *FSD1*, or *ZIP2* (Fig. 4), displayed higher expression levels at 0h than at 12h. We have recently proposed a model developed for deciphering and understanding the spatiotemporal codes for oscillatory gene regulation (Peñarrubia *et al.*, 2015). Frequently, the expression of both the activator (i.e. SPL7) and its target genes in the response to an input (i.e. Cu deficiency) that triggers the activator function is subjected to circadian regulation. Hence, its expression will oscillate with similar periods but with defined phase differences. The oscillatory expression of the activator will act as a multiplicative factor on the target mRNA synthesis when in phase with its targets, since the phase synchrony will contribute to ‘constructive interference’, in analogy to the physical concept of waves adding up their amplitudes when in phase. As a result, the transcription of a target gene that oscillates with the same phase as the activator will increase. On the other hand, the target genes that oscillate in antiphase with the activator will display only a moderately increased and somewhat distorted transcriptional response. In agreement with this model, most of the SPL7-dependent best regulated genes show a close phase synchrony with *SPL7* (Table 1).

Since HY5 has been shown to interact with SPL7 at promoter regions comprising the respective *cis*-regulatory elements G-box and GTAC (Zhang *et al.*, 2014) (Supplementary Fig. S5 at *JXB* online), this TF could be considered a putative mediator of the effect of light on SPL7-dependent Cu deficiency responses. However, under our experimental conditions, the expression of *SPL7* and the Cu deficiency markers used in this work (*COPT2*, *FSD1*, *YSL2*, and *ZIP2*) remains mostly unchanged in the *hy5* mutant (Supplementary Fig. S4). HY5 affects Cu homeostasis through the co-regulation of miR408 and, whereas *FSD1* is co-regulated by both HY5 and SPL7 TFs, *COPT2* is not (Zhang *et al.*, 2014). Taken together, our results and previous data by Zhang *et al.* (2014) suggest that in addition to HY5, other mechanisms must exist to control light dependence of Cu deficiency responses.

Phytochromes and the functional state of chloroplasts have been suggested to participate in a new retrograde Fe sensor-dependent route, which constitutes a central oscillator loop (Salomé *et al.*, 2013). Moreover, *COPT2* has been found to function in both Cu and Fe homeostasis (Perea-García *et al.*, 2013), and red light and phytochromes, at least, partially affect Cu deficiency responses (Fig. 5A, B). On the other hand, the integrated role of blue light and the clock through GI is well established (Kim *et al.*, 2007; Sawa *et al.*, 2007), and here we show that Cu down-regulates *GI* expression in a dose-dependent manner (Fig. 3). Importantly, *GI* induction under Cu deficiency does not appear to occur in the *spl7* mutant (Fig. 6) which is in accord with *GI* displaying two putative GTAC elements in its promoter region. These results reinforce the role of Cu status in connection with the Arabidopsis circadian clock and suggest a complex and multilevel effect of light on Cu deficiency responses.

The SPL7-dependent regulation by the Cu status of plasma membrane COPTs, such as that encoded by *COPT2*, has led to the hypothesis of the existence of a self-regulatory feedback loop of Cu on expression of its own transporters that can cause oscillation in COPT expression which could result in a cycling Cu concentration (Peñarrubia *et al.*, 2010). Accordingly, we show here that the Arabidopsis *COPT2* transcripts are circadian regulated (Fig. 3). In addition, it was also previously shown that *COPT1* and *COPT2* are among the circadian-regulated nutrient transporters (Haydon *et al.*, 2011).

If a Cu oscillation takes place, the subcellular location of the cycling Cu fluxes remains unknown. In this respect, the suggestion that SPL7-mediated Cu deficiency stress responses can be perceived in the endoplasmic reticulum lumen (Garcia-Molina *et al.*, 2014) opens up new possibilities. Light and circadian regulation of ion channels and nutrient transporters remains unresolved. It has been proposed that they regulate downstream targets to spread light and circadian signalling further which, in turn, can provide feedback on the central oscillator (Ko *et al.*, 2010; Haydon *et al.*, 2011). Thus, if we consider this the other way around (i.e. how Cu homeostasis affects the circadian function), Cu down-regulates the expression of key genes in the circadian clock and photoperiod perception such as *LHY*, *CCA1*, *HY5*, and *GI* (Fig. 6). In addition, *GI* and *PHYA* expression is reduced in the *spl7* mutant under Cu deficiency (Fig. 6). These results indicate that Cu affects the circadian and photoperiodic functions in plants.

*COPT1* ^*OE*^ and *spl7* mutants show circadian clock-related alterations, such as the amplitude of cotyledon movement (Fig. 7). This is in agreement with a role for Cu homeostasis in circadian clock outputs. Although *COPT1* ^*OE*^ and *spl7* displayed a reduced growth compared with the WT, perhaps accounting for part of the reduction in the amplitude of cotyledon movements, the severity of this reduction, especially in the case of *COPT1* ^*OE*^ where it decreased to such an extent that it could no longer be recognized by the BRASS software, points to altered Cu homeostasis affecting the circadian outputs. In accordance with this, SPL7 has been shown to bind the *CCA1* promoter (Zhang *et al.*, 2014) and its expression is down-regulated by Cu (Fig. 6; Andrés-Colás *et al.*, 2010). These findings indicate that Cu homeostasis can serve as both circadian clock input and output pathways. The role that transition metals play in redox control and mitochondrial and chloroplastic electron transport, and their involvement in oxidative stress generation, are noteworthy (Ravet and Pilon, 2013). Since oxidative stress seems to play a key role in circadian rhythm generation (Lai *et al.*, 2012), the interconnection between metals and oxidative stress can be the basis of the effects that metal transport have on the molecular clock. Interconnections between Cu homeostasis and the circadian rhythms have been reported in organisms other than plants. Recently, Cu availability has been shown to affect the circadian clock of suprachiasmatic nuclei (SCN) in humans, where it appears to modulate the phase through glutamate signalling (Yamada and Prosser, 2014).

The central circadian oscillator is considered an integrator of external environmental cycles and of internal rhythms involving soluble, cytosolic ions and metabolites that can act as both inputs and outputs within the overall circadian network. The global dynamic nature of the connection between diurnal rhythms and oscillating intracellular biochemistry is depicted as time wheels (Perea-García *et al.*, 2010; Pruneda-Paz and Kay, 2010). The aim of interconnected feedback loops is to increase the clock robustness and inertia under weak environmental cycles and extend oscillations to the entire cell metabolism, resulting in enhanced plant fitness.

In summary, these results indicate that light and the circadian clock greatly affect Cu deficiency responses related to gene expression in which SPL7 acts as a positive regulator. Diurnal and circadian effects are exerted in differential and multiple ways that may include factors, such as HY5, GI, and others. In this way, temporal integration adapts plant development to light/dark cycles and oscillatory nutrient homeostasis for optimal plant performance under challenging deficient environmental conditions.

## Supplementary data

Supplementary data are available at *JXB* online.

Figure S1. Luciferase activity at different Cu concentrations.

Figure S2. *FSD1*, *COPT2*, and *LUC* gene expression at different Cu contents.

Figure S3. Regulation of *CSD1* and *CCS1* gene expression by Cu and light conditions.

Figure S4. Regulation of gene expression by Cu in the *hy5* mutant.

Figure S5. Sequence analysis of the *COPT2*, *FSD1*, and *SPL7* promoters for putative HY5 and SPL7 recognized sites.

Table S1. Analysis of the peak time values under different circadian and diurnal oscillatory conditions for the genes regulated under Cu deficiency conditions.

Table S2. Primers used for cloning the *FSD1* and *COPT2* promoters.

Table S3. Oligonucleotides used in sqPCR.

Table S4. Oligonucleotides used in qPCR.

Supplementary Data
