# Supplementary material for: Modulation of copper deficiency responses by diurnal and circadian rhythms in Arabidopsis thaliana
Source: J Exp Bot. 2015 Oct 29;67(1):391–403. doi: 10.1093/jxb/erv474 (PMC4682440; doi:10.1093/jxb/erv474)
Supplement: Supplementary Data [file supp_67_1_391__index.html]

Modulation of copper deficiency responses by diurnal and circadian rhythms in Arabidopsis thaliana — Modulation of copper deficiency responses by diurnal and circadian rhythms in Arabidopsis thaliana — Supplementary Data 

# Modulation of copper deficiency responses by diurnal and circadian rhythms in *Arabidopsis thaliana*

## Supplementary Data

Data files

- supplementary\_figures\_S1\_S5-Tables\_S1\_S4.pdf - Supplementary Data
