# Supplementary material for: Modulation of copper deficiency responses by diurnal and circadian rhythms in Arabidopsis thaliana
Source: J Exp Bot. 2015 Oct 29;67(1):391–403. doi: 10.1093/jxb/erv474 (PMC4682440; doi:10.1093/jxb/erv474)
Supplement: Supplementary Data [file supp_erv474_supplementary_figures_S1_S5-Tables_S1_S4.pdf]

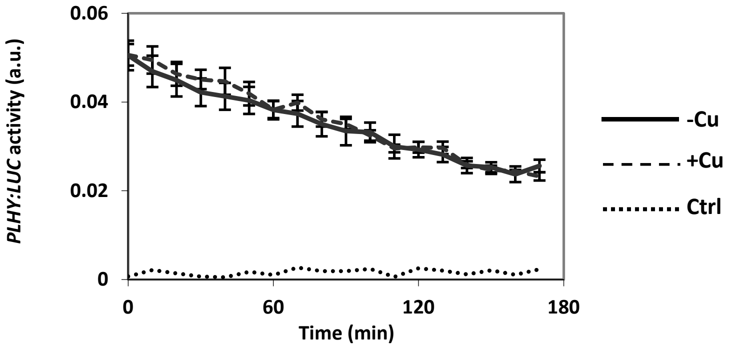

Fig. S1. Luciferase activity at different Cu concentrations. Total protein was extracted from 7 day-old *PLHY:LUC* seedlings grown under Cu deficiency (½ MS; -Cu). Kinetic measurements were performed after adding 10 µM $CuSO_4$ (Cu excess; +Cu) and a constant amount of D-luciferin (0.3 mg/mL). The activity was recorded every 10 min. Extract without substrate was used as a negative control (Ctrl). a.u.= arbitrary units.

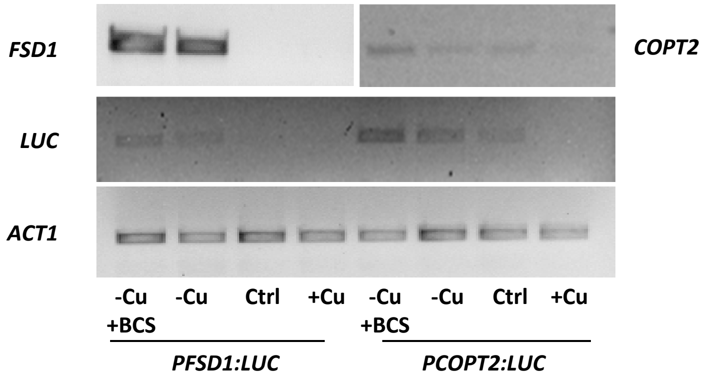

Fig. S2. *FSD1, COPT2* and *LUC* gene expression at different Cu contents. Six day-old *PFSD1:LUC* and *PCOPT2:LUC* seedlings were grown in severe Cu deficiency (½ MS with 50 µM BCS; -Cu + BCS), Cu deficiency (½ MS; -Cu), sufficiency (½ MS with 1 µM µM $CuSO_4$, Ctrl) or in Cu excess (½ MS with 10 µM µM $CuSO_4$; +Cu) in LDHC cycles. Samples were collected at 4 h after the onset of the light cycle and *LUC* and *FSD1* gene expression were analyzed by sqPCR. *ACT1* expression was used as loading control.

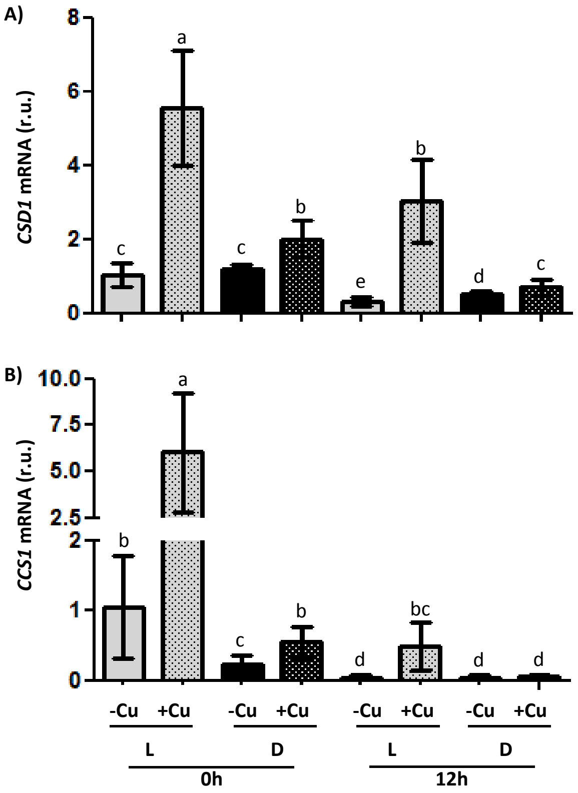

Fig. S3. Regulation of *CSD1* and *CCS1* gene expression by Cu and light conditions. The relative expression was determined in the same conditions shown in Fig. 4. Means with a different letter are significantly different ($P < 0.05$). r.u. = relative units.

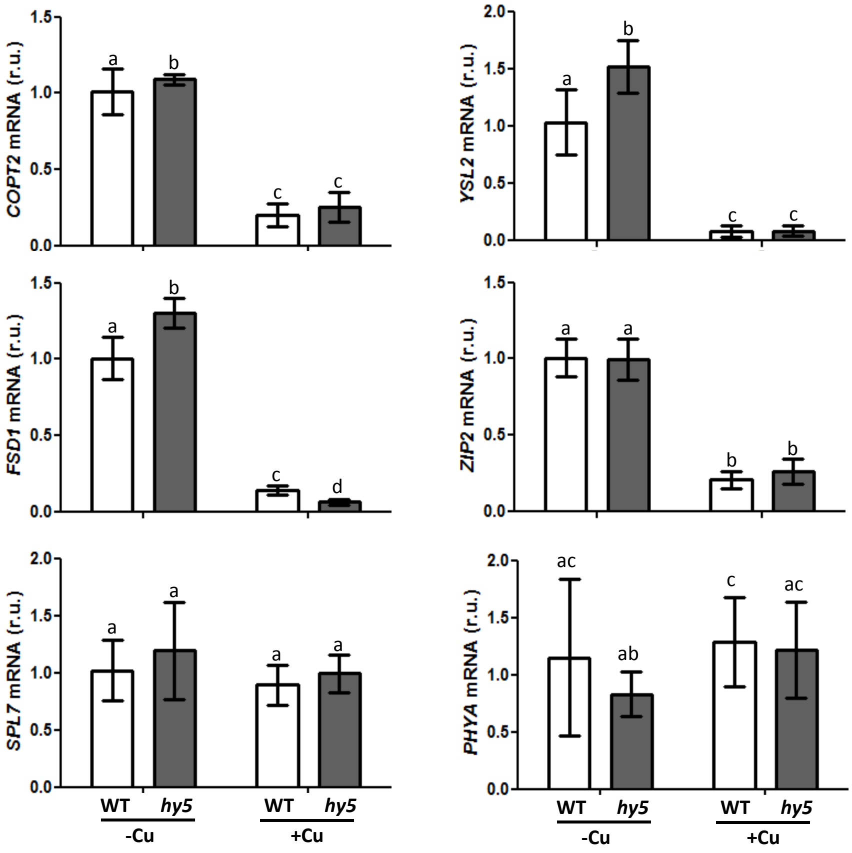

Fig. S4. Regulation of gene expression by Cu in the *hy5* mutant. The relative expression of the *COPT2*, *FSD1*, *SPL7*, *YSL2*, *ZIP2* and *PHYA* genes was determined by qPCR in 7 day-old WT seedlings grown under Cu deficiency (½ MS; -Cu, white bars) or Cu excess (½ MS with 10 µM $CuSO_4$; +Cu, grey bars). The *UBQ10* gene was used as reference. mRNA levels are expressed as relative units (r.u.). The bars represent the mean ± SD of 3 replicates. Means with a different letter are significantly different ($P < 0.05$).

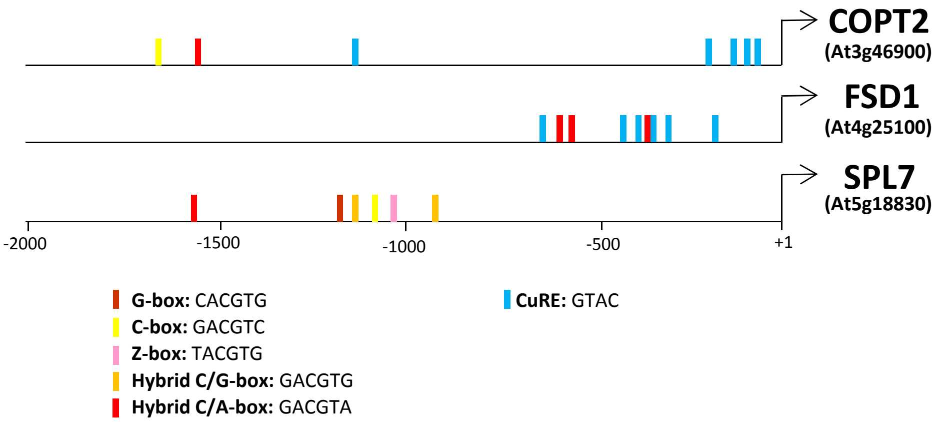

Fig. S5. Sequence analysis of the *COPT2*, *FSD1* and *SPL7* promoters for putative HY5 and SPL7 recognized sites. Positions of the *cis* regulatory elements are marked in different colors as indicated in figure legend. Putative CuRE elements bound by SPL7 (Yamasaki *et al.,* 2009) and different HY5 bound elements (Song *et al*., 2008) are shown.

Table S1. Analysis of the peak time values under different circadian and diurnal oscillatory conditions for the genes regulated under Cu deficiency conditions. The locus MIPS code (http://www.arabidopsis.org) and the phases under different oscillatory conditions (http://diurnal.mocklerlab.org) are shown. D: dark, L: light, H: hot (22˚C), C: cold (12˚C). Letters between brackets mean entrainment conditions before constant conditions.

		Circadian					Diurnal						
**Gene**	Locus	**DD(DDHC)**	**LL23(LDHH)**	**LL12(LDHH)**	**LL(LDHC)**	**LL(LLHC)**	**LDHH_SM**	**LDHH_ST**	**longday**	**shortday**	**LDHC**	**LLHC**	**Peak**
COPT2	AT3G46900		20	22				21	0	22			21.8
FSD1	AT4G25100		6					1					3.5
SPL7	AT5G18830						19			22	23	22	21.5
ZIP2	AT5G59520									19			19
CSD1	AT1G08830	20		6		23							0.3
CCS	AT1G12520	23					22	21	23	18			21.4
LHY	AT1G01060	0	22	1	1	2	0	23	1	23	23	2	0.2
CAS	AT5G23060		21	23		3	2	23	2	1	2	2	0.7
YSL2	AT5G24380		19	21						4		21	22.3
SMC6B	AT5G61460								1	10		18	18
IRT1	AT4G19690			1	4				23				1.0
ACA1	AT3G52720		19		22	23	1	0	21	21	1		22.5
TIR-NBS-like	AT3G04210							16	21		7		22.7
CYP83A1	AT4G13770			7		22	8	8	9	6	8	23	5.4
GSTF11	AT3G03190						8	10		6		0	6
BCAT4	AT3G19710						7	8	7	6		3	6.2
UGT78D2	AT5G17050	21	19	22	23	23		19		5	22	21	22.1
EXPA8	AT2G40610		4	6	6			0		23	5	8	4.0
Aux resp	AT1G29440		1	5	3	3	3	1	3	23	3	6	2.6
COPT1	AT5G59030						21	21	22	22			21.5
CCH	AT3G56240							0		20			22
CSD2	AT2G28190		15	7		23						0	17.3

Table S2. Primers used for cloning the *FSD1* and *COPT2* promoters. The restriction site is indicated *in italics* in the sequence.

Name	Sequence (5'-)
COPT2-1200F-HindIII	GTGGTGTTA***AAGCTT***TTGCAACAA
COPT2-ATG-R-HindIII	CAT***AAGCTT***GATACTAATGTTAATAGGGTTTATGAT
FSD1 HindIII F	TTT***AAGCTT***GGAGTGAAGCTATATATA
FSD1 HindIII R	TTT***AAGCTT***AGCAGCCATTCTTTGTAATTGA

Table S3. Oligonucleotides used in sqPCR.

Gene Name	Sequence (5′-)
ACTINARA1A	GGCGATGAAGCTCAATCCAAAC
ACTINARA1B	GGTCACGACCAGCAAGATCAAGACG
FSD1 rt F1	GCTTCAAGTGCTGTCACC
FSD1 rt R1	CAAGCCAGGCCCAGCCAG
LUC-F	GGAAGACGCCAAAAACAT
LUC-R	GTTTTGTCACGATCAAAGGA
COPT2-F	ACGTGTCAGTGGCTCAACC
COPT2-R	GACGGCGGAAGAAGCTCGGCGG

Table S4. Oligonucleotides used in qPCR.

Gene Name	Sequence (5'-)	
	forward	reverse
COPT2	CCTTTCGTATTTGGTGATGCT	AAACACCTGCGTTAAAGGAC
CCS	TCTCCACGTCTCTTGGGACTTT	AGCTGAGGCATGGCTCGAT
CSD1	CATCATTGGTCTCCAGGGCT	GACCTCCTTATTACATCAAT
FSD1	ACCGAAGACCAGATTACATA	TGGCACTTACAGCTTCCCAA
SPL7	CAGGCAGACTGTTCACCAGA	AGTTTGACGGGACCTGAATG
UBQ10	TAATCCCTGATGAATAAGTGTTCTAC	AAAACGAAGCGATGATAAAGAAG
ZIP2	CGCTTGGAGAAACCTATGGA	CGACACCTATGGGACTCGAT
HY5	GTTTGGAGGAGAAGCTGTCG	TCTTGCTTGCTGAGCTGAAA
PHYA	GTTACAAAACCTGGGCTGGA	ATGTTGGCCATGTACTGCAA
YSL2	TCTTATAAATGGATTTCATACTA	AATGCCCAAAAGAAACTCAAA
LHY	TCGGCCTCTTCTTCACAGTT	ACACCCGAGCAATTCTCATC
CCA1	GTTGCAGCTGCTAGTGCTTG	GAAGATCGAGCCTTTGATGC
TOC1	TGATCTCCCAATGGCTAAGG	ACTTCGTCTTGCCTCGACAT
GI	GCATCTAGTTGCTGGCCTTC	AGCTCGAAGGAGTTCCACAA
